# Young Nursing Student’s Knowledge and Attitudes about Contraceptive Methods

**DOI:** 10.3390/ijerph17165869

**Published:** 2020-08-13

**Authors:** Sebastián Sanz-Martos, Isabel María López-Medina, Cristina Álvarez-García, María Zoraida Clavijo-Chamorro, Antonio Jesús Ramos-Morcillo, María Mar López-Rodríguez, Ana Fernández-Feito, Silvia Navarro-Prado, María Adelaida Álvarez-Serrano, Laura Baena-García, María Ángeles Navarro-Perán, Carmen Álvarez-Nieto

**Affiliations:** 1 Department of Nursing, Faculty of Health Sciences, University of Jaén, Campus Las Lagunillas, 23071 Jaén, Spain; ssanz@ujaen.es (S.S.-M.); cagarcia@ujaen.es (C.Á.-G.); calvarez@ujaen.es (C.Á.-N.); 2 Department of Nursing, School of Nursing and Occupational Therapy, University of Extremadura, 10003 Caceres, Spain; zoraidacc@unex.es; 3 Department of Nursing, Faculty of Nursing, University of Murcia, Campus Espinardo, 30100 Murcia, Spain; 4 Department of Nursing, Physiotherapy and Medicine, University of Almería, 04120 Almería, Spain; mlr295@ual.es; 5 Department of Medicine, University of Oviedo, 33003 Oviedo, Spain; fernandezfana@uniovi.es; 6 Department of Nursing, Faculty of Health Sciences of Melilla, University of Granada, 52005 Melilla, Spain; silnado@ugr.es; 7 Department of Nursing, Faculty of Health Sciences of Ceuta, University of Granada, 51001 Ceuta, Spain; adealvarez@ugr.es; 8 Department of Nursing, Faculty of Health Sciences, University of Granada, 18071 Granada, Spain; lbaenagarcia@ugr.es; 9 Faculty of Nursing, Catholic University of Murcia, Guadalupe de Maciascoque, 30107 Murcia, Spain; manavarro2@ucam.edu

**Keywords:** nursing students, young health, sexuality, contraceptive methods

## Abstract

Purpose: Adolescence is considered a period in which individuals are particularly at risk of negative consequences related to sexual health. Increased knowledge levels have traditionally been used as an indicator of the effectiveness of educational programs, but attitudes are not addressed and are a key element for the success of such programs. The aim of this study is to determine the level of knowledge and attitudes toward the use of contraceptive methods among nursing students. A multicenter cross-sectional study was carried out. In total, 2914 university students (aged 18–25 years) enrolled in the study. Participants completed two validated scales to measure knowledge level and attitudes toward contraceptive use. Nursing degree students who received training about contraceptives obtained a success rate of over 70%, compared to 15.3% among students who had not received such training (*p* < 0.001). The mean attitude score was 43.45 points (10–50), but there were no significant differences in terms of student training (*p* = 0.435), although they were significantly higher among students who used contraceptives at first or last sexual intercourse (*p* < 0.001). There was a significant weak correlation between the level of knowledge and attitudes toward the use of contraceptives. An adequate level of knowledge about sexuality and contraceptive methods does not correspond to positive attitudes toward their use, although having an excellent attitude toward contraceptive use is related to their use during youth and adolescence.

## 1. Introduction

The World Health Organization (WHO) states that comprehensive education about sexuality has a central role in preparing young people for safe, productive, and fulfilling lives in a world where Human Inmunodeficiency Virus (HIV), sexually transmitted infections (STIs), unintended pregnancy, gender-based violence, and gender inequality still pose a serious risk to well-being [1]. Countries increasingly recognize the importance of equipping young people with the knowledge, attitudes, and skills to make responsible choices in their lives, particularly in a context where they have greater exposure to sexually explicit materials through the Internet and other media [2].

Provision of the sexual and reproductive health information and services that young people need is critical to preventing major problems [3]. Scientific evidence shows that sex education programs can lead to delayed sexual intercourse, reduced unprotected sex, fewer sexual partners, and increased contraceptive use [4]. However, in many countries there is a lack of adequate formal and planned training on sexuality and contraceptive methods (Sex&CM) [1]. The survey on Sexual Health and Contraception in Spanish Youth in 2019 highlights this lack of training and information on sexuality and contraception, as 68.5% of young people surveyed considered that they did not have enough training [5].

The university is a context in which young people learn to manage their intimate relationships and sexuality [6], but formal training in sexuality is not a reality, and these young people have few decision-making tools for certain scenarios [7]. Although WHO promotes educational programs as a preventive measure for individuals regardless of whether they are sexually inactive or active [2], educational interventions on Sex&CM in young populations are scarce, and the studies carried out show a predominantly low level of knowledge [6,8,9,10,11,12]. The increase in the level of knowledge about Sex&CM in adolescents has been widely used as the only variable to confirm the effectiveness of educational programs for the prevention of unwanted pregnancies [13] and other programs aimed at avoiding other consequences—such as STIs—of risk behaviors in young people [6]. 

Knowledge is the basis for directing attitudes, and both knowledge and attitudes are essential for reducing risky sexual behavior [6]. Educational programs must include the variables of knowledge, skills, and attitudes. Traditionally, educational programs have been delivered using a vertical teaching methodology and have based their effectiveness on increasing the level of knowledge without addressing attitudes toward use, but the latter is a key element in program success [14]. It is important to note that the knowledge young people acquire about Sex&CM is based on the best scientific evidence. The main sources of information among young people are the peer group and the Internet, which provide empirical knowledge based on their own experiences [15] or knowledge based on low-quality scientific sources [5,16].

Previous research on the attitudes of university students toward sexuality and the use of contraceptives has shown the importance of having reliable sources of information, which allow students to obtain an optimal level of knowledge and develop attitudes based on scientific evidence [17,18]. Lack of knowledge about contraceptives and their use is associated with a high risk of unwanted pregnancy, and ambivalent attitudes toward pregnancy and abortion increase the likelihood of unsafe contraceptive practices [9]. To protect young people from the risks associated with sexual and reproductive health, it is therefore important to determine their level of knowledge, attitudes, behaviors, and needs [3]. Previous research to evaluate the level of knowledge and attitudes has measured both variables without using validated scales that allow a valid and reliable measurement [6,8,9,10,11,12,17,18]. It is thus hypothesized that the level of knowledge about Sex&CM determines young people’s attitudes toward Sex&CM. The objective of this study was to determine the level of knowledge about Sex&CM to explore attitudes on these issues among nursing students and to evaluate the correlation between both variables. 

## 2. Materials and Methods

### 2.1. Design

The study was designed as a multicenter cross-sectional study.

### 2.2. Sample and Setting

This study used a convenience sample of undergraduate nursing students, between the ages of 18 and 25 (inclusive), from seven Spanish universities.

### 2.3. Data Collection

Data were collected from students in the four courses of the nursing degree (September 2018–June 2019) by trained persons, in sessions of 10–15 min’ length during the large group classes. Self-administered data collection notebooks in paper format included the following:

- Sociodemographic variables: gender, age, university to which they belonged, academic course, having received training on Sex&CM during the nursing degree, having a partner at the time of study, source of information used to obtain information on Sex&CM, source of information desired to obtain information on Sex&CM, self-perception of their level of knowledge on Sex&CM, and self-perceived knowledge gap.
- Level of knowledge about Sex&CM: measured through the SexContraKnow-Instrument scale, validated in Spanish and consisting of 15 items with three answer options (True/False, Do not know, No answer). The scale showed a reliability of 0.99 for items and 0.74 for people [19]. The score range is between 0 and 15, and the level of knowledge is categorized as excellent (≥90%), very good (70–89%), good (55–69%), insufficient (30–54%), and poor (≤29%).
- Attitudes toward contraceptive use: measured through a subscale previously validated with a Cronbach’s alpha of 0.71 [20]. Responses were evaluated using a Likert scale with values from 1 to 5 (1 strongly disagree and 5 strongly agree). The score range is between 10 and 50 and the attitude level is categorized as excellent (≥90%), good (70–89%), and insufficient (≤69%).
- Sexual activity variables: having had penetrative sex, age at first sexual encounter, use of any contraceptive method at first intercourse and contraceptive method used or reason for not using any, use of any contraceptive method at last intercourse and contraceptive method used or reason for not using any.
- Level of knowledge about family planning centers (FPCs): First, using a dichotomous question (Yes/No), participants were asked whether they knew about these centers. If the answer was yes, they had to respond to six more statements, with three answer options (True/False, Do not know, No answer). If the number of correct answers was greater than 50%, they were considered to know about FPCs. We made this section of the questionnaire ad hoc for this research and it was revised by experts, but its validity and reliability have not been assessed.

### 2.4. Data Analysis

Descriptive statistics for all data and questionnaire scores were calculated. Bivariate analysis was performed, establishing as dependent variables the score on the knowledge and attitudes scales and as independent variables the sociodemographic variables, variables regarding sexual relations, and the variable concerning knowledge about FPCs. The normality of the distribution was evaluated using analysis of the histogram, asymmetry, kurtosis, and the Kolmogorov–Smirnov test. The contrast was made using non-parametric tests. The size of the effect was calculated using the biserial correlation of ranges of Glass (r) for the contrasts where the Mann–Whitney U test was used, while for the contrasts using the Kruskal–Wallis test, the epsilon square statistic (*ε*^2^) and the biserial correlation of ranges in the back-testing contrasts were used [21].

Multivariate analysis was performed using a multiple linear regression model. The linearity and independence of the residues were checked using the Durbin–Watson statistic, with values between 1.5 and 2.5 determined as acceptable values for independence. The homoscedasticity and normality of the standardized scores of the residues were determined by analysis of the histogram and a scatter plot. The model’s goodness of fit was calculated using the value of R^2^.

All analyses were performed with the statistical program SPSS 24.0 (Version 24.0, IBM: Armonk, DA, USA), and a value of *p* < 0.05 was established as the level of statistical significance. For post hoc contrasts between variables with more than two categories, the significance level was corrected using the Bonferroni test (*α*/Contrasts).

**Figure 1:**
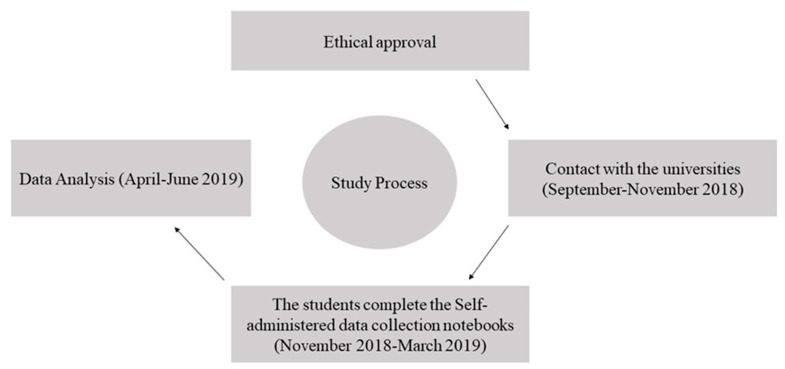
Shows the process of this study.

For completing the study we have followed 4 stages that can see in Figure 1.

### 2.5. Ethical Considerations

This study was approved by the Institutional Review Board of the University of Jaén (ABR.17/9). The objectives of the research and the non-obligatory nature of participation were explained to the students. There was no penalty if they declined to participate. In addition, participants were asked to provide written consent, and all of their questions were answered. Confidentiality of personal data was guaranteed.

## 3. Results

### 3.1. Sample Characteristics

The initial sample consisted of 3408 undergraduate students. After applying the inclusion criteria, 7 were eliminated for not signing the informed consent, 55 for not completing the questionnaire, 347 for being older than 25 years, and 79 for being younger than 18 years at the time of the survey. Finally, the sample consisted of 2914 undergraduate students in nursing degree programs, with a mean age of 20.28 ± 1.955 years (Table 1).

At the time of the survey, 80.2% of the participants claimed to be sexually active, with the average age of sexual debut being 16.55 (SD: 1.506). The main sources of information used on Sex&CM were the Internet and health professionals via educational talks. The main self-perceived knowledge gap was sexuality and forms of non-coital sexual relations, followed by contraceptive methods, with no statistically significant differences between groups (*p* > 0.05; Table 1). As for the use of the contraceptive methods, a statistically significant decrease in the most recent sexual relations is observed with respect to the first (66.7% vs. 73.3%, respectively; χ^2^ = 42.870; *p* < 0.001) the main reason being the improvisation of the sexual relations in both cases. There was also a greater use of condoms during first sexual relations (71.8%) compared to the most recent (43.4%; χ^2^ = 34.768; *p* < 0.001), and a significant increase in the use of the contraceptive pill, which was about 1% during first sexual relations and 20.1% during most recent sexual relations (χ^2^ = 26.639; *p* < 0.001).

### 3.2. Training in Sex&CM

The average score for the knowledge level scale was 8.17 ± 2.907. The items on sexuality and the male condom had the highest success rate (1, 2, 3, 7), while the main gaps in knowledge were found in the items on the contraceptive patch (10, 11, 12) and the vaginal ring (13, 14, 15). The mean score for the attitude scale was 43.45 ± 5.099. Item 2 (Using condoms is a hassle because it reduces sensation) scored the lowest on the scale, while items 3 (If I hooked up with someone other than my partner, I would use condoms to avoid possible infections) and 9 (I prefer to use contraceptives before finding myself with an unwanted pregnancy) scored the highest (Table 2).

The students who had not received training (61.3%) had insufficient or poor knowledge, but this percentage significantly decreases among trained students (24.2%; χ^2^ = 44.748; *p* < 0.001). More than half of the trained students (52.3%) had more than 70% correct answers (very good or excellent level of knowledge), but this was reduced to only 15.3% among untrained students (Table 3). Table 4 shows the bivariate contrasts for the knowledge scale. Participants who had received training during the nursing course obtained significantly higher scores than those who had not, although this difference was weak (Z = −10.644, *p* < 0.0001; *r* = 0.24).

The source of information used for training on Sex&CM showed significant effect at the bivariate level. In a posteriori contrasts, it was found that the participants who were trained by health personnel obtained significantly higher scores, with respect to other sources used such as: the Internet (Z = −6.797, *p* < 0.001, *r* = 0.41); friends (Z = −7.848, *p* < 0.001, *r* = 0.37); or their parents (Z = −4.736, *p* < 0.001, *r* = 0.38), all differences being weak. Finally, the type of contraceptive method used during most recent intercourse gained statistical significance, with the level of knowledge being higher among participants who used the contraceptive pill or the vaginal ring than in participants who used the male condom (Z = −12.570, *p* < 0.001, *r* = 0.32 and Z = −6.702, *p* < 0.001, *r* = 0.22, for the contraceptive pill and the vaginal ring, respectively) or withdrawal (Z = −3.507, *p* < 0.001, *r* = 0.33 and Z = −4.386, *p* < 0.001, *r* = 0.23, for the contraceptive pill and the vaginal ring, respectively). No significant differences were found among participants using the birth control pill or the vaginal ring (Z = −3.061, *p* = 0.002).

Overall, only 5.9% of students had poor attitudes, with almost half of the students (48.8%) showing excellent attitudes (Table 3). Females scored significantly higher on the attitude scale (Z = 6.638, *p* < 0.001, *r* = 0.41). The source of information used for training on Sex&CM showed a significant effect at the bivariate level, with participants who were trained by health personnel obtaining significantly higher scores compared to those of others relying on sources such as the Internet (Z = −3.795, *p* < 0.001, *r* = 0.45) or their friends (Z = −4.619, *p* < 0.001, *r* = 0.43), both of which were weak differences. Moreover, participants who used a contraceptive method during first or more recent intercourse had significantly higher scores (Z = 7.343, *p* < 0.001, *r* = 0.34 and Z = −17.679, *p* < 0.001, *r* = 0.22, respectively). Finally, the type of contraceptive method used during most recent intercourse gained statistical significance, with the most positive attitudes appearing among participants who used a male condom (Z = −7.149, *p* < 0.001, *r* = 0.17), the contraceptive pill (Z = −6.801, *p* < 0.001, *r* = 0.17), or the vaginal ring (Z = −5.048, *p* < 0.001, *r* = 0.18) compared to those who used withdrawal. There were no statistically significant differences between participants using the birth control pill, vaginal ring, or male condom (*p* > 0.0083; Table 4).

### 3.3. Factors Influencing Knowledge and Attitudes toward Contraceptive Use

For the scale of knowledge about Sex&CM, 12 variables were introduced. The model consisted of five predictors, with a high correlation between these and the scale score (0.562) explaining the model at 31.4% of variance. The ANOVA for the multiple regression model showed a statistically significant relationship (F = 151.132; *p* < 0.001). The collinearity of the model according to the model-conditioning index obtained a value of 22.135, which can be considered moderate collinearity. The Durbin–Watson statistic obtained a value of 1.745, so the residues were considered independent. The variable *having received training on sexuality and contraceptive methods during the nursing course* was the one that obtained the highest linear correlation coefficient for the knowledge level variable, that is, the correlation between both variables was moderate (0.432). The other variables included in the model obtained weak correlations, although all were significant (*p* < 0.05) (Table 5).

For the attitude scale, 14 variables were initially introduced in the regression model. Again, a model formed of five predictors was obtained, with the correlation between the five predictors and the scale score being high (0.419) and explaining the model at 17.4% of variance. The ANOVA for the multiple regression model also showed a statistically significant relationship (F = 99.460; *p* < 0.001). The collinearity was also moderate (16.693), and the Durbin–Watson statistic was 1.877, so the residues were also considered independent. The variable *use of any contraceptive method during most recent intercourse*, obtained the highest linear correlation coefficient for the knowledge level variable, with the correlation between both variables being weak–moderate (0.355). The other variables included in the model obtained weak correlations, although all were significant (*p* < 0.05) (Table 5).

## 4. Discussion

The hypothesis is partially supported by our research data, as there is a significant weak correlation between the level of knowledge and attitudes toward contraceptive use among nursing students. Similar results were found by Kgosiemang and Blitz [22] for the emergency contraceptive pill (*p* < 0.001). However, in our study, the significant effect was not maintained in the subsequent multivariate analysis, although the level of knowledge is an important element for the development of positive attitudes toward the use of contraceptive methods, coinciding with two previous researches [6,23]. The weak correlation between the level of knowledge and the score on the attitude scale indicates the existence of other variables involved in the development of attitudes toward contraception that should be investigated qualitatively.

### 4.1. Knowledge Level about Sex&CM

Knowledge level about Sex&CM among young nursing students was good and supported by excellent attitudes; however, it was insufficient or poor among students who had not received any training during the degree, which coincides with the results of other research on young university students [8,10,11,12]. In addition to this knowledge deficit, the age of initiation of sexual relations was 16.5 years, similar to that found in other research [5,24,25,26]. Therefore, the need for more training in early stages, before the onset of sexual relations to prevent negative consequences related to their experience of sexuality should be emphasized [23,27] through education programs on sexual and reproductive health and consultation units in universities [28].

In Spain, the use of the Internet is a majority practice among young people between the ages of 16 and 24 (99.2% in men and 99% in women [29]), and it is also the main source of information used for training in Sex&CM, surpassing other sources that had previously been significant [5,25,30]. This change may be due to the embarrassment students may feel in addressing these issues, with the Internet being an easily accessible and apparently private source [31]. Coinciding with Munakampe et al. [32] and as reported by the Spanish Society for Contraception [5], the Internet remained one of the main sources of information considered for future occasions, mainly among men, while women preferred more personal sources of information, such as health professionals or their parents.

Students who obtained information from healthcare providers had significantly higher levels of knowledge and excellent attitudes toward contraceptive use, demonstrating the importance of having quality information to improve such knowledge and attitudes [17,18,33]. However, while health professionals are such a source of quality information, there is a need to reach out to young people so that they do not feel inhibited on a topic that is usually embarrassing to them [34]. The creation of mobile applications could be a key element in providing the sources of information used by young people, while offering them the advantage of obtaining high-quality information anonymously and immediately, allowing improvements to the level of knowledge and attitudes [4,35].

Overall, statistically significant gender differences were found for the level of knowledge about Sex&CM, with the latter being higher among women than was found by two studies [8,28]. It was, however, in line with Muanda et al. [34], who also found that women are more knowledgeable about contraceptive methods other than the male condom. Women showed significantly more positive attitudes toward the use of contraceptive methods, coinciding with Muanda et al. [34], which makes us consider the need to evaluate attitudes in terms of gender, as recommended by Heras and Lara [36]; these authors found that men were more interested in aspects of the sexual relationship, while women showed a greater interest in contraceptive issues to prevent adverse consequences.

### 4.2. Attitudes toward Contraceptive Use

Students who used a contraceptive method at first and most recent intercourse scored higher on the attitude scale. At the multivariate level, this statistical significance was maintained, and these variables were predictive of good scores on the attitude scale [37]. In line with previous studies [5,24,25,38], the male condom is the method most often chosen for first and most recent sexual relations, although for the latter other contraceptive methods such as the contraceptive pill and vaginal ring appear more frequently, suggesting greater foresight and planning for such sexual encounters and also a greater awareness, at a later age, of the risks of not using any method at all. In addition, the level of knowledge among students who used a hormonal contraceptive method during the most recent intercourse was significantly higher than that of those who used a male condom, with the self-education of young people in responsible decision-making being important [5].

The main reason that led participants not to use any contraceptive method during first and most recent sexual encounters was the improvised nature of the relations, but for the most recent encounter, there was a remarkable increase of other response options: they did not want to use any or contraceptives took away pleasure. Improvisation of sexual intercourse as a cause for not using contraception coincides with one of the main barriers to male condom use identified by Peterson et al. [39]. Other use-related barriers such as loss of pleasure associated with condom use [39], difficulty obtaining contraceptives [32], or lack of knowledge about different contraceptive options [17,32,33,39], were little used by the students in this study, especially for most recent intercourse.

Finally, knowledge of FPCs was significantly related to knowledge level and attitudes. Such centers offer confidential information, can improve the level of knowledge and enable the development of positive attitudes based on scientific knowledge. However, most students were unaware of the existence of FPCs and, to a lesser extent (20.3%) expressed gaps in knowledge about places to go for the information and services offered by these centers. Shame in obtaining information or the belief that they will be judged are important barriers to accessing these centers among young people [40]. On the other hand, although men were more exposed to risks, most planning services are oriented toward women (family planning and gynecological care). It would therefore be desirable to make sexual and reproductive health services more attractive by offering flexible schedules and adapting information to the gender of the student [6].

The main limitation of this research is that, as a self-administered questionnaire, the participants could have copied the right answers from another partner and the results could have been overestimated. Due to the voluntary nature of the completion of the questionnaire, the motivation to participate in the research is a key element, and there could be participants who, due to their self-perception of low knowledge, decided not to participate. Furthermore, because it is considered a taboo subject, participants may not have been totally honest when answering questions about sexual relations, and the variable referring to the use of contraceptive methods during first and most recent sexual encounters may be overestimated. The section of the questionnaire about FCPs lacks a validation process, so we must be cautious in interpreting the relationships. To finish, the sample was mainly made up of women, so care must be taken when interpreting the results.

## 5. Conclusions

Having received training on Sex&CM during the nursing course statistically increased students’ level of knowledge. Therefore, it is essential to have formal education on sexuality and affectivity that can prevent future situations of machismo, violence, or sexism, as well as unwanted pregnancies and the spread of STIs. The attitudes of the young do not depend only on the level of knowledge about contraceptive methods, there are other variables that influence them. Positive attitudes toward the use of contraceptive methods have a significant effect on their use during the first and most recent sexual encounters and, thus, influence young people’s behavior. A good source of information can prevent knowledge gaps and clarify misconceptions, as well as foster skills to support empowerment, positive values and attitudes, and healthy behaviors.

Henceforth, both health managers and trainers should consider that attitudes in sexuality and contraception education are an essential element for the acquisition of a good level of knowledge and the adoption of healthy sexual behaviors. Likewise, the use and knowledge of FPCs also leads to increased knowledge and positive attitudes toward FPCs. Finally, in future researches we will include young people with both university and non-university backgrounds, as well as students from non-health disciplines. We will carry out the validation process for the scale about the FPCs. Moreover, the use of qualitative methodology allows us to know the reality of young people in matters of sexuality.

## Figures and Tables

**Table 1:** Sociodemographic variables of the sample.

Variable	Categories	Frequency
Gender	Men	571 (19.6%)
	Woman	2343 (80.4%)
University	University of Jaén	352 (12.1%)
	University of Murcia	549 (18.8%)
	University of Extremadura	117 (4.1%)
	Catholic University of Murcia	648 (22.2%)
	University of Oviedo	202 (6.9%)
	University of Granada	717 (24.6%)
	University of Almería	239 (11.3%
Having partner	Yes	1720 (59%)
	No	1194 (41%)
Received training on sexuality and contraceptive methods during the nursing course	Yes	1459 (50.1%)
	No	1455 (49.9%)
Source of information used to obtain information on sexuality and contraceptive methods	Internet	1374 (47.2%)
	Healthcare professionals	768 (26.4%)
	Friends	544 (18.7%)
	Parents	166 (5.7%)
	Talks on sexual and reproductive health	41 (1.4%)
	Own or friends’ experience	21 (0.7%)
Source of information desired to obtain information on sexuality and contraceptive methods	Internet	1267 (43.5%)
	Talks on sexual and reproductive health	1562 (53.6%)
	TV Campaigns	69 (2.4%)
	Others	16 (0.5%)
Self-perception of their level of knowledge on sexuality and contraceptive methods	Bad	57 (2%)
	Regular	1007 (34.5%)
	Good	1850 (63.5%)
Self-perceived knowledge gap	Sexuality and forms of non-coital sex	932 (32%)
	Contraceptive methods	860 (29.5%)
	Places to request information	230 (7.9%)
	Places to obtain contraceptive methods	591 (20.3%)
	I do not need more information	289 (9.9%)
	Sexually transmitted diseases	12 (0.4%)
Had penetrative sex	Yes	2336 (80.2%)
	No	578 (19.8%)
Use of any contraceptive method at first intercourse	Yes	2137 (73.3%)
	No	199 (6.9%)
Contraceptive method used at first intercourse	Male Condom	2092 (71.8%)
	Contraceptive pill	23 (0.8%)
	Withdrawal method	22 (0.7%)
Reason for not using any contraceptive method at first intercourse	Improvised sexual intercourse	132 (4.5%)
	Were not planning to use it	44 (1.5%)
	Reduce pleasure	14 (0.6%)
	Shame to get a contraceptive method	9 (0.3%)
Use of any contraceptive method during most recent intercourse	Yes	1939 (66.5%)
	No	397 (13.7%)
Contraceptive method used during most recent intercourse	Male Condom	1266 (43.4%)
	Contraceptive pill	586 (20.1%)
	Vaginal ring	49 (1.7%)
	Withdrawal method	38 (1.3%)
Reason for not using any contraceptive method during most recent intercourse	Improvised sexual intercourse	146 (5%)
	Were not planning to use it	128 (4.4%)
	Reduce pleasure	123 (4.3%)
Knowledge about FPCs	Yes	949 (32.6%)
	No	1965 (67.4%)

Data expressed by frequencies and percentages from the total sample (*n* = 2914). FPCs: family planning centers.

**Table 2:** Descriptive statistics for scales of knowledge and attitudes (*n* = 2914).

Variable	M	SD
SexContraKnow-Instrument		
There is a risk of pregnancy when you have unprotected sex in the 2 days before or after ovulation.	0.95	0.23
The male condom is safe if placed just before ejaculation, even if penetration has occurred previously.	0.97	0.17
The “calendar method” (calculating the fertile period for not having sexual intercourse within this period) is effective in preventing pregnancy.	0.80	0.40
When you start taking the birth control pill, it is effective from day one.	0.70	0.46
Hormonal contraceptive methods of birth control (for example, the birth control pill or vaginal ring) are recommended for adolescents.	0.28	0.45
When one forgets to take the contraceptive pill at the correct time, it can be taken without a loss of effectiveness as long as no more than 12 h have passed since the original time.	0.59	0.49
The “dual contraceptive method” consists of the simultaneous use of a barrier contraceptive method (e.g., male condom) and a hormonal contraceptive method (e.g., contraceptive pill).	0.71	0.45
If the contraceptive pill is started after the 5th day of the menstruation cycle, using another contraceptive method for one week is recommended.	0.44	0.50
The pattern of taking the contraceptive pill is one pill per day from the 1st day of the cycle for 21 days, with a week of rest (placebo pills may be taken during this week).	0.72	0.45
The contraceptive skin patch must be applied on the first day of the menstruation cycle.	0.24	0.43
Replacement of the birth control skin patch should be done only when the patch detaches itself.	0.37	0.48
The contraceptive skin patch should be placed on the buttocks, lower abdomen, upper back, or outer arm.	0.41	0.49
For colocation of the vaginal ring, it is necessary to see a specialist.	0.50	0.50
During sexual intercourse, the vaginal ring can be removed for 2 h without risk of pregnancy.	0.12	0.32
The vaginal ring should be left in place for 21 days, followed by a week of rest.	0.40	0.49
**Attitudes Scale**		
I would not have intercourse without using birth control.	3.78	1.28
Using condoms is a hassle because it reduces feeling.	3.42	1.30
If I hooked up with someone other than my partner, I would use condoms to avoid possible infections.	4.82	0.63
Using contraceptives allows for safer and more pleasurable relationships.	4.20	0.97
I do not want to use any birth control.	4.44	0.98
I would not mind carrying condoms, even if others thought badly of me.	4.31	1.04
If you only make love once in a while, it is not worth using contraceptives.	4.66	0.84
Sexual relations with contraceptives lose their grace.	4.20	1.08
I prefer to use contraceptives before finding myself with an unwanted pregnancy.	4.82	0.64
Contraceptive methods are so unsafe that they are not worth using.	4.77	0.64

M: mean; SD: Standard deviation.

**Table 3:** Category scales for level of knowledge and attitudes toward use of contraceptive methods.

Categories	Knowledge		Attitudes	
	Trained	Not Trained	Trained	Not Trained
Excellent	220 (15.1%)	25 (1.7%)	721 (48.2%)	701 (48.2%)
Very Good	543 (37.2)	198 (13.6%)	—	—
Good	343 (23.5%)	340 (23.4%)	648 (44.4%)	673 (46.3%
Insufficient	303 (20.8%)	638 (43.8%)	90 (6.2%)	81 (5.6%)
Poor	50 (3.4%)	254 (17.5%)	—	—

**Table 4:** Bivariate contrasts for the SexContraKnow-Instrument scale and the Attitude toward Contraceptive use scale.

Variable	Knowledge M ± SD	Contrast	Attitudes M ± SD	Contrast
Gender		Z = −10.644 **		Z = −6.638 **
Men	7.03 ± 2.71		41.98 ± 5.96	
Women	8.49 ± 2.92		43.80 ± 4.80	
Age		Rho = 0.412 **		Rho = −0.064 **
Have received formation during the nursing degree				
Yes	9.51 ± 2.73	Z = −24.552 **	43.46 ± 5.19	Z = −0.780
No	6.89 ± 2.52		43.43 ± 5.01	
Source of information				
Internet	8.04 ± 2.93	χ^2^ = 75.406 **	43.33 ± 4.88	χ^2^ = 31.547 **
Healthcare professionals	8.92 ± 2.96		44.10 ± 4.77	
Friends	7.72 ± 2.86		42.70 ± 5.58	
Parents	7.80 ± 2.62		43.64 ± 6.01	
Talks on sexual and reproductive health	8.20 ± 2.57		44.27 ± 6.47	
Own or friend’s experience	8.24 ± 2.23		43.62 ± 4.35	
Self-perception of their level of knowledge on sexuality and contraceptive methods				
Good	8.71 ± 2.90	χ^2^ = 165.153 **	43.53 ± 5.17	χ^2^ = 3.840
Regular	7.35 ± 2.78		43.30 ± 4.90	
Bad	6.63 ± 2.48		43.35 ± 6.16	
Having had intercourse				
Yes	8.59 ± 2.86	Z = −14.485 **	43.27 ± 5.07	Z = −4.846 **
No	6.65 ± 2.72		44.16 ± 5.18	
Age of first intercourse		Rho = −0.047 *		Rho = 0.094 **
Use of any contraceptive method at first intercourse				
Yes	8.58 ± 2.83	Z = −0.066	43.49 ± 4.01	Z = −7.343 **
No	8.60 ± 3.11		40.88 ± 5.11	
Contraceptive method used at first intercourse				
Male condom	8.58 ± 2.84	χ^2^ = 1.543	43.56 ± 4.91	χ^2^ = 13.421 **
Contraceptive pill	9.09 ± 2.05		42.57 ± 5.85	
Withdrawal method	8.45 ± 2.22		38.05 ± 8.67	
Use of any contraceptive method during most recent intercourse				
Yes	8.63 ± 2.84	Z= −1.554	44.11 ± 4.60	Z = −17.679 **
No	8.35 ± 2.96		39.17 ± 5.25	
Contraceptive method used during most recent intercourse				
Male condom	8.04 ± 2.80	χ^2^ = 189.252 **	44.34 ± 4.52	χ^2^ = 53.395 **
Contraceptive pill	9.78 ± 2.50		44.05 ± 4.35	
Vaginal ring	10.71 ± 1048		43.67 ± 5.21	
Withdrawal method	8.18 ± 2.59		37.84 ± 5.70	
Knowledge about FPCs				
Yes	9.31 ± 2.82	Z= − 14.132 **	44.28 ± 4.80	Z = −6.371 **
No	7.67 ± 2.83		43.04 ± 5.19	
Knowledge–Attitudes		Rho = 0.05 **		Rho = 0.05 **

* *p* < 0.05; ** *p* < 0.01. M: mean SD: Standard deviation.

**Table 5:** Model values for the scale of attitudes toward the use of contraceptive methods.

	Variable	Beta	*p*	Correlation Coefficient
Knowledge about sexuality and contraceptive methods	Having received training on sexuality and contraceptive methods during the nursing course	0.379	<0.001	0.436
	Contraceptive method used at the last intercourse	0.180	<0.001	0.244
	Gender	0.192	<0.001	0.204
	Knowledge about FPCs	0.160	<0.001	0.253
	Self-perception of their level of knowledge on sexuality and contraceptive methods.	0.132	<0.001	0.210
Attitudes toward contraceptive use	Use of any contraceptive method during most recent intercourse	0.349	<0.001	0.355
	Gender	0.141	<0.001	0.153
	Use of any contraceptive method at first intercourse	0.084	<0.001	0.091
	University	−0.084	<0.001	−0.092
	Knowledge about FPCs	0.068	<0.001	0.102
